# 40 years of forest dynamics and tree demography in an intact tropical forest at M’Baïki in central Africa

**DOI:** 10.1038/s41597-024-03577-6

**Published:** 2024-07-06

**Authors:** Fabrice Bénédet, Sylvie Gourlet-Fleury, Félix Allah-Barem, Fidèle Baya, Denis Beina, Guillaume Cornu, Luc Dimanche, Émilien Dubiez, Éric Forni, Vincent Freycon, Frédéric Mortier, Dakis-Yaoba Ouédraogo, Nicolas Picard, Vivien Rossi, Olivia Semboli, Yves Yalibanda, Olga Yongo-Bombo, Adeline Fayolle

**Affiliations:** 1 Forêts et Sociétés, Université de Montpellier, CIRAD, Montpellier, France; 2 CIRAD, Forêts et Sociétés, Montpellier, France; 3 Institut Centrafricain de la Recherche Agronomique, Bangui, Central African Republic; 4 Ministère des Eaux, Forêts, Chasse et Pêche, Bangui, Central African Republic; 5 Université de Bangui. Faculté des Sciences. Laboratoire de Biodiversité Végétale et Fongique, Bangui, Central African Republic; 6 Fonds de Développement Forestier, Bangui, Central African Republic; 7 Institut national de Recherche Forestière, Brazzaville, Republic of the Congo; 8 Université Marien Ngouabi, Brazzaville, Republic of the Congo; 9 Independent researcher, Athens, Greece; 10 GIP ECOFOR, Paris, France; 11 Centre d’Études et de Recherche en Pharmacopée et Médecine Traditionnelle Africaine, Université de Bangui, Bangui, Central African Republic; 12 Gembloux Agro-Bio Tech, Université de Liège, Gembloux, Belgium

**Keywords:** Forest ecology, Biodiversity, Tropical ecology, Ecosystem ecology, Forestry

## Abstract

A vast silvicultural experiment was set up in 1982 nearby the town of M’Baïki in the Central African Republic to monitor the recovery of tropical forests after disturbance. The M’Baïki experiment consists of ten 4-ha Permanent Sample Plots (PSPs) that were assigned to three silvicultural treatments in 1986 according to a random block design. In each plot, all trees with a girth at breast height greater than 30 cm were spatially located, numbered, measured, and determined botanically. Girth, mortality and newly recruited trees, were monitored almost annually over the 1982–2022 period with inventory campaigns for 35 years. The data were earlier used to fit growth and population models, to study the species composition dynamics, and the effect of silvicultural treatments on tree diversity and aboveground biomass. Here, we present new information on the forest stand structure dynamics and tree demography. The data released from this paper cover the three control plots and constitute a major contribution for further studies about the biodiversity of intact tropical forests.

## Background & Summary

In 1982, the Technical Center for Tropical Forests (CTFT, now part of the International Center for Agronomic Research – CIRAD) established a vast silvicultural experiment in the forested part of the Central African Republic, near the town of M’Baïki^1,2^. The so called M’Baïki experiment was part of the project “Application de la Recherche à la mise en valeur des Ressources Forestières et Cynégétiques – 1978/1985” funded by the “Fonds d’Aide et de Coopération”. This project aimed to develop management methods for natural forests and addressed the following main questions. (1) What is the future of tropical logged forests? (2) Will the logged forests recover enough valuable trees to allow a sustainable timber tree production?

The M’Baïki experiment consists of ten 4-ha permanent sample plots (PSPs) that were assigned to three silvicultural treatments according to a random block design: control (three plots), logging (three plots), and logging plus thinning (four plots). Four main objectives were assigned to these PSPs: (1) to study the growth of valuable tree species, depending on simplified treatments; (2) to study forest dynamics and tree demography (comprising mortality and recruitment) depending on these treatments; (3) to synthesize knowledge gained on the valuable tree species ecological requirements; (4) to estimate treatments costs and balance them with production gains. This data paper covers the three control plots (12 ha in total) and the data released constitutes a major contribution for further studies about the biodiversity of intact forests in the region and across the global tropics.

In each 4-ha plot, all trees over 30 cm girth at breast height (gbh) were spatially located, numerated, measured, and determined botanically to the species or morphospecies level. Since 1982, gbh (converted into diameter at breast height, dbh, in the database), standing deaths, treefalls, and newly recruited trees (measured in the field over 30 cm gbh, but recruited in the database over 10 cm dbh) have been monitored annually except in 1997, 1999, 2001, 2013, 2014 and 2016. The three control plots thus provide longitudinal data (measurements repeated over time) on growth, mortality and recruitment for 10,885 trees, and overall biodiversity dynamics in intact forests, over 40 years.

The M’Baïki plots are among the earliest and largest PSPs that have been set up in tropical forests to monitor long-term changes in forest dynamics according to textbooks^3,4^. With the exception of the comparable forest experiment that was established at the same period in French Guiana^5^, the other PSPs are smaller or were established more recently, in Suriname^6^, across the Amazon^7^ and Atlantic forests^8^, in Uganda^9^ and across tropical Africa^10^, in Malaysia^11^, India^12^, and in Papua New Guinea^13^. PSPs are often large in area (>1 ha) and heavily instrumented but have few spatial repetitions and local ecological representativeness, which makes them complementary to management and national forest inventories^14,15^. Their specificity is to provide longitudinal data over time, making them all the more valuable as they are older^16^. PSPs were originally established in the 1970s in tropical forests to assess the impact of logging and design silvicultural treatments that comply with sustainable forest management. They supported the development of growth and yield or forest dynamics models^5,17–21^.

The amount of data progressively accumulated over time in PSPs has made them particularly relevant to address questions beyond silviculture and management, including ecological questions related to the different facets of tree biodiversity and forest structural complexity and the effect of climate change on tropical forest ecosystems^22^. PSPs scattered across the Amazon region revealed a decline in net biomass change over the 1983–2011 period due to a clear increase in mortality, while productivity also increased, albeit to a lesser extent^23^. This significant trend was attributed to accelerated growth and shortened lifespans, indicating accelerated life cycles, which aligns with greater climate variability. While this trend has been observed in the Amazon, it remains to be demonstrated for African forests^24^ but long-term data is lacking and specifically the last decade 2010–2020 is not covered by published studies. An increase in aboveground carbon stocks in live trees between 1968 and 2007 have been reported in intact African forests^25^, and this increase is particularly evident in the control plots of M’Baïki^16^. However, it is still necessary to clarify the main factors involved (forest ageing, CO2 fertilization) and determine whether the pronounced drying trend recorded in Africa^26^ has an impact on forest dynamics^27^ and biodiversity since altered phenology and threats on the megafauna have been observed in Gabon^28^.

PSPs data has been collated within plot networks at the regional, continental and now global scale^22,29–31^ and combined with other forest inventory plots or unstructured data (like species occurrences) to address global ecological questions and biodiversity issues. Recent studies examined the relationship between forest biodiversity and productivity^32,33^, the number of tree species in the tropics^34^ and on Earth^35^, invasion by non-native tree species^36^, or the forest carbon potential^37^ thanks to new approaches in data analysis like machine learning or artificial intelligence allowing to deal with massive but heterogeneous data.

Though representing the second largest block of tropical forests after the Amazon, the Congo Basin is usually under-represented in studies across the global tropics^38^ and the ongoing decline in carbon sequestration in trees observed for the Amazon^23^ has not been observed yet for Africa^24^. More importantly longitudinal data over time are extremely important to disentangle long-term dynamics from transitory processes^39^. For these reasons, the 40-yr dynamics of the M’baïki forest experiment (with inventory campaigns for 35 years) represents a gold mine to test theoretical questions, in addition to the practical applications for which the silvicultural treatments (logging and thinning) were originally designed^16,40^. In addition, located at the northern edge of the Congo basin, nearby the forest-savanna transition^41^, this site is thus a good candidate to observe early signs of transition in response to climate changes, and specifically increase in drought stress due to rising temperatures predicted for Africa, and already observed in Gabon^28^.

## Methods

### Study site

#### Location

**Fig. 1:**
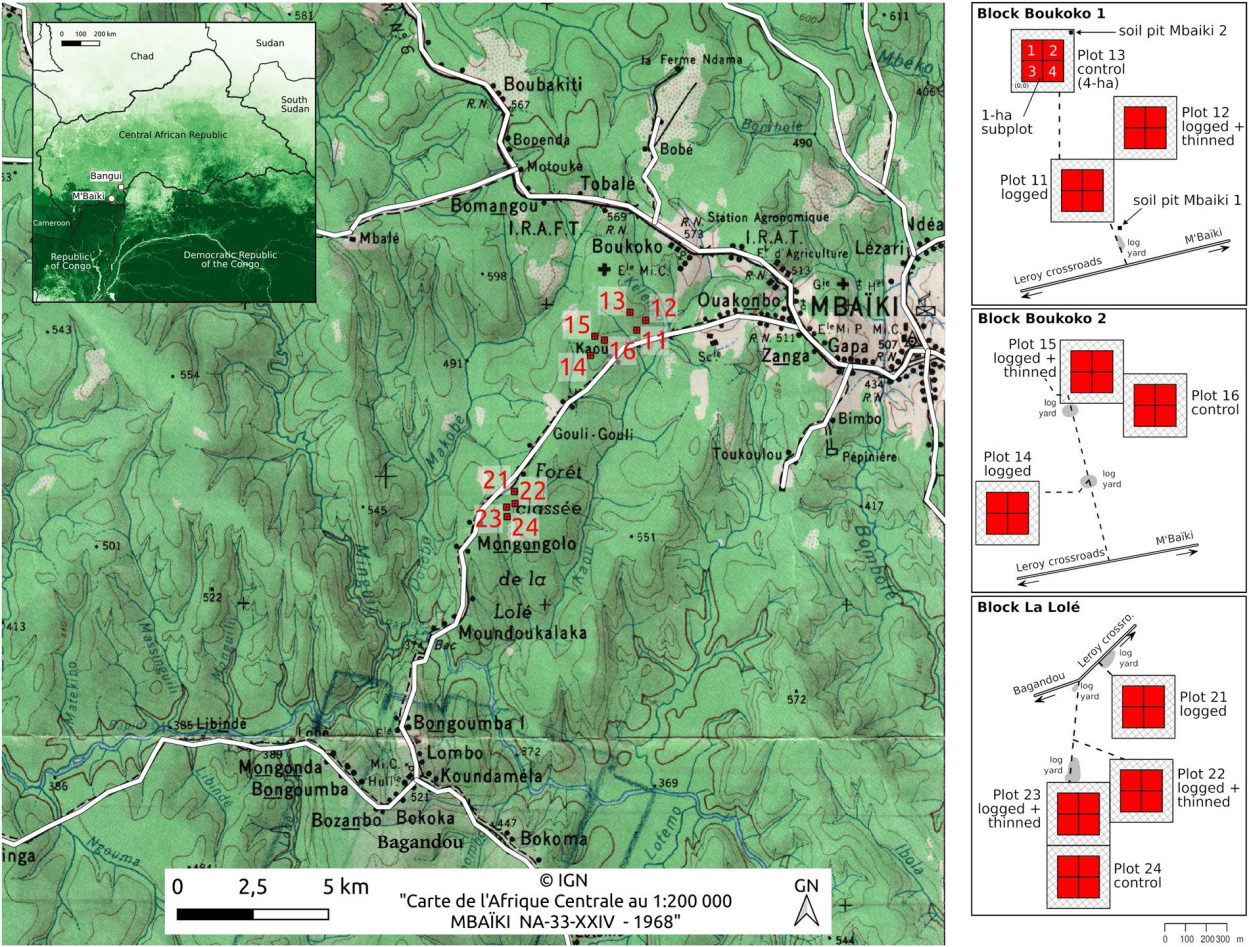
Location of the PSPs (in red) nearby the city of M’Baïki in the Central African Republic at the northern edge of the Congo basin as visible on the tree cover map^72^ shown as inset. The same silvicultural treatments (control, logging, and logging + thinning) have been applied to the 4 ha-plots (in red) and to the 50 m-wide buffer zones (hashed) in the three experimental blocks (Boukoko 1, Boukoko 2 and La Lole). Each 4 ha-plot is divided into four 1 ha subplots.

The city of M’Baïki is located 120 km in the southwest of Bangui in the Lobaye province of the Central African Republic (3.54°N 17.56°E). The forest perimeter retained for the installation of PSPs in 1982 corresponds to the forests of Boukoko (2,500 ha) and La Lolé (2,900 ha) located circa 10 km south of M’Baïki in the Bagandou direction (Fig. 1). The land use prior to plot establishment was dense forest in the 1980s, and dating back at least to the 1960s according to the IGN map (Fig. 1) and even to the 1920s according to the book “Voyage au Congo”, in which André Gide describes the area as densely forested, with extremely tall trees^42^. The presence of a logging company in the surroundings of M’Baïki is mentioned in the same book, but industrial logging was restricted to the East of M’Baïki and South of Bangui in the 1960s^43^. To our knowledge, these forests were never logged before and were considered representative of the Lobaye forests according to a forest survey made in 1952 by the local forest service. The PSPs are now included in the conservation area of the forest exploitation permit (“PEA 171”) granted to a logging company, the “Société Centrafricaine de Déroulage” (SCAD) with a management plan covering the 2005–2034 period. The company was highly impacted by the coup d’état of March 2013 but it has a new owner since 2021.

#### Climate

The climate was early described as Guinean forested of the Oubangian type, with mean annual rainfall between 1600 and 1700 mm^1^. The mean annual rainfall is 1651 mm over the 2007–2012 period according to the rain gauge of the Boukoko meteorological station located nearby and 1537 mm over the 2015–2018 period according to Climate Hazards Group InfraRed Precipitation with Station data (CHIRPS) for a mean annual temperature of 24.8 °C over the 2015–2018 period according to the Climate Forecast System Reanalysis (https://www.climateengine.org/). The climate is highly seasonal with a pronounced dry season lasting from 3 to 6 months according to the rain gauge data, and from 3 to 4 months according to CHIRPS.

#### Topography, substrate and soils

The topography is undulated in the area but the PSPs were installed on a relatively flat terrain (550 m asl., Fig. 1). The geological substrate consists of Precambrian schists, sandstones and quartzites and a total of 12 soil units were identified and mapped in the PSPs according to the CPCS classification and the soil color^44^. One soil type, the “slightly to moderately desaturated ferrallitic soils” dominates (88% of the plot area), with the majority being “typical modal or deep soils” (49% of the plot area) and corresponding to Haplic Ferralsols according to the international soil classification^45^. These soils are characterized by a sandy clay loam texture, there are acidic (pH 4.9), and show low CEC values and available P concentration according to the two original soil profiles^44^ (Table 1). Several charcoals were observed at 30, 60 and 110 cm depth in the soil profile Pr4, with the vast majority of them at 60 cm, and one small charcoal was observed at 60 cm in the soil profile Pr7 (V. Freycon, pers. obs.). Two other soil profiles (soil pit Mbaiki 1 and 2, Fig. 1) were earlier described up to 6 m in a study about the root distribution of *Entandrophragma cylindricum*^46^. The other soils recognized in the PSPs are Pisoplinthic Ferralsols (21% of the plot area), Pisoplinthic Plinthosols (18%) and Leptosols (12%).

**Table 1:** Physical and chemical properties of two soil profiles for the ‘slightly to moderately desaturated ferrallitic soils, typical modal or deep soils’ (i.e., Haplic Ferralsols) that dominates in the M’Baïki PSPs^44^.

Profile	Depth (cm)	Clay (%)	Silt (%)	Sand (%)	pH	C (%)	N (‰)	P Bray2 (mgkg^−1^)	CEC (cmo_*c*_kg^−1^)
Pr4	0–15	18	3	79	4.7	1.0	0.9	3.7	3.9
	30	25	4	71	4.9	0.7	0.6	2.5	4.4
	70	25	5	70	4.8	0.4	0.3	2.5	3.2
	110	30	3	67	4.8	0.3	0.2	2.0	4.1
Pr7	0–15	22	4	74	4.7	0.9	0.8	2.9	4.2
	40	24	4	72	4.8	0.5	0.4	2.0	4.2
	90	32	4	64	4.8	0.4	0.3	1.8	4.1

#### Vegetation

The PSPs are located at the northern edge of the Congo basin (Fig. 1), nearby the forest-savanna transition^41^. The landscape is dominated by lowland tropical forest, but the prolonged existence of open vegetation, encompassing savannas and agricultural complexes along roads, as well as in proximity to cities and villages, should be acknowledged (Fig. 1). The tropical forests in the area are of the semi-deciduous type, characterized by the abundance and diversity of species of the Myristicaceae, Cannabaceae, Meliaceae, Fabaceae families^47^. The abundance of deciduous taxa in the area was indeed confirmed by management inventory data performed by logging companies^48,49^. Past human activities probably influenced forest structure and composition in the M’Baïki PSPs, but the area remains to be sampled for human artifacts. There is a growing consensus that most forests in central Africa were gardened prior to European colonisation^50^ but we currently lack evidence for the M’Baïki PSPs. Charcoals were observed in soils but with low frequency and abundance in comparison to other sites in Cameroon^51^. The presence of Kapok trees (*Ceiba pentandra*) with immense buttresses around the city of M’Baïki in the 1920s^42^ is a good indicator of past human activities, but in contrast to other places, such as Gabon^52^, there is no map of ancient villages available for the area, and known archaeological sites are located further East, near the Ubangi river^50^.

#### Experimental design

The M’Baïki experiment consists of ten 4-ha PSPs (40 ha in total) that were assigned to three silvicultural treatments: control (3 plots), logging (3 plots), and logging plus thinning (4 plots) according to a random block design applied in the three experimental blocks: Boukoko 1 (3 plots, 12 ha), Boukoko 2 (3 plots, 12 ha) and La Lolé (4 plots, 16 ha). Logging and thinning (girdling followed by poisoning of some undesirable trees to promote the growth of valuable trees) were applied in 1986. Around each 4-ha plot, a 50 m buffer zone was installed to prevent edge effects (Fig. 1). Silvicultural treatments were thus applied over 9 ha but tree measurements, localisation, and identification were restricted to the central 4-ha plots, which were further subdivided into 1-ha subplots. This data paper covers the three control plots (12 ha in total).

### Tree measurements

#### Overview of the measurements

**Fig. 2:**
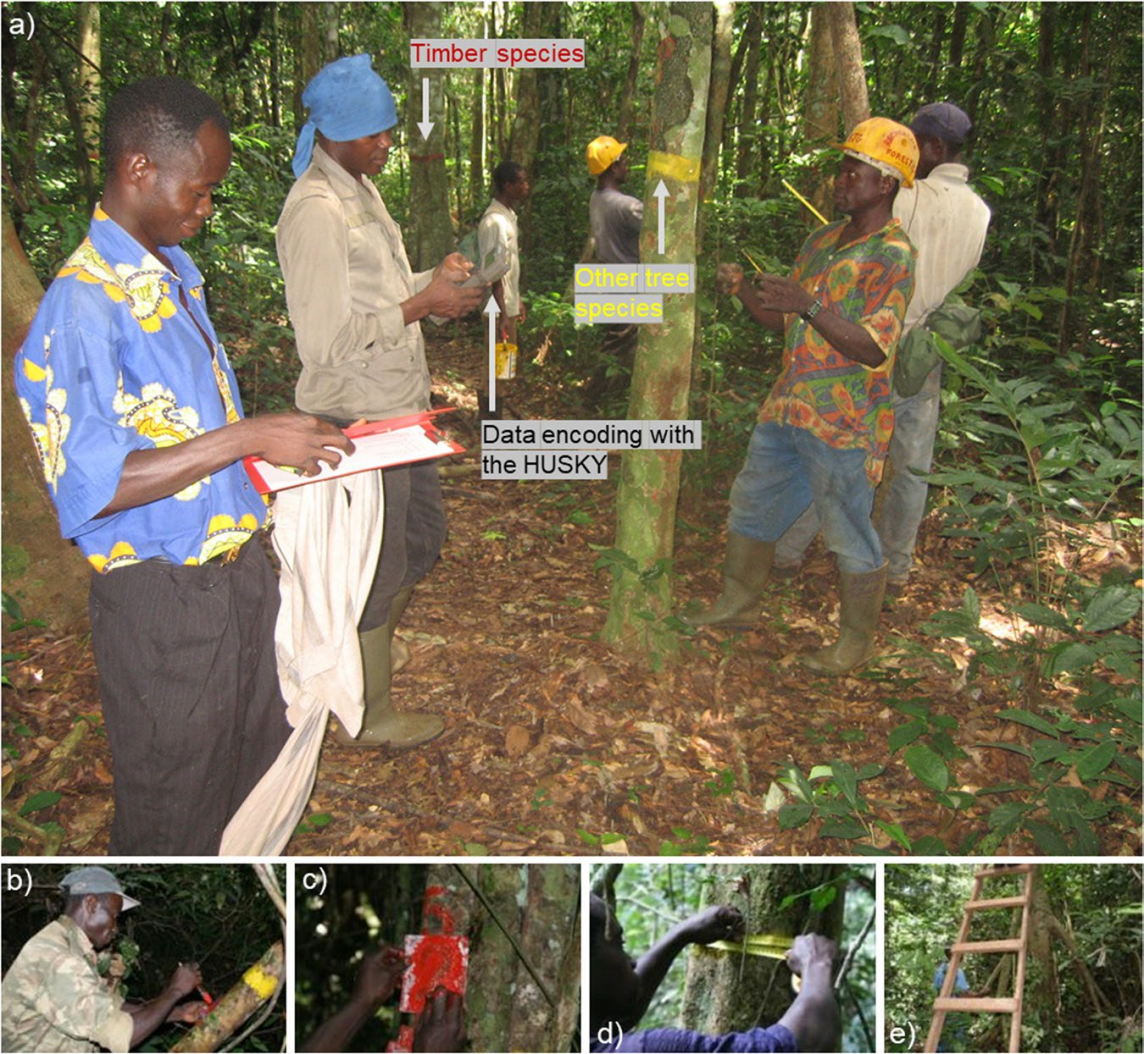
Field work during inventory campaigns in the M’Baïki PSPs. Trees are spatially located in the 4-ha plots (**a**) and numbered in the 1-ha subplots (**b,c**), their girth is measured at breast height (**d**) and 50 cm above deformation, up to 4.50 m high with a ladder for extremely irregularly shaped trees (**e**). © E. Dubiez for (**a**) in 2007 and A. Fayolle for (**b**–**e**) in 2011. The height of measurement (hom) is painted on the trees, in red for the trees belonging to the 36 valuable timber species or species complexes recognized at installation (Table 2), and in yellow for all other trees (**a**).

In each 4-ha plot, all trees with a girth at breast height (gbh) greater than 30 cm were spatially located, numbered, measured, and determined botanically to the species or morphospecies level (Fig. 2). In the database, gbh were converted into diameter at breast height (dbh) and the inventory threshold generally used is 10 cm dbh but the data from 9.5 cm dbh (corresponding to 30 cm gbh) were also used in some studies^53,54^. Since 1982, gbh, mortality (standing deaths, treefalls), and newly recruited trees measured in the field over 30 cm gbh, but recruited in the database over 10 cm dbh, were monitored annually except in 1997, 1999, 2001, 2013, 2014 and 2016. Forest inventory data are thus available for 35 years over the 1982–2022 period. Since 1998, the inventory campaigns have been led by F. Baya, who supervises the inventory of trees measured at breast height (with the help of three to five field workers) while a subteam equipped with a ladder is responsible of the trees measured above breast height (two field workers). The inventory of the ten 4-ha plots is made between April and June and lasts between one month and one month and a half (depending on weather conditions). Each field campaign is followed by a data quality control.

#### Spatial coordinates

All trees with gbh greater than 30 cm are located within the 4-ha plots from the (0;0) reference point located at the South-West of the plot (Fig. 1). This point is marked on the ground with a 50 cm-deep pit, so as the other plot corners and the centre of the 1 ha subplots^19^. The spatial coordinates of trees range from 0 to 200 m within this reference system. Since the (0;0) reference point was georeferenced with the average value of GPS track maintained during several hours at the point, the trees can be mapped in any coordinate system, and the projected UTM 35 N is classically used.

#### Tree numbering

For practical reasons (high tree density, >500 ha^−1^ before the silvicultural treatments), trees are numbered within the four 1-ha subplots. Thus the tree id is composed of the number of the 4-ha plot (plots 11, 12, and 13 for the Boukoko 1 block, plots 14, 15, and 16 for the Boukoko 2 block, and plots 21, 22, 23, and 24 for the La Lolé block, Fig. 1), the number of the 1-ha subplot (1, 2, 3, 4 for all plots), and the tree number within the 1-ha subplot. The latter is painted on the tree (Fig. 2b,c). Readability is checked during each inventory campaign, and painting is refreshed when needed.

#### Girth measurements

The tree girth is measured using a classical tape (not a forester tape) and the value is rounded at 0.5 cm in the field. In the database, girth values are converted into diameters with a 0.1 cm rounded value.

The height of measurement (hom) equivalent to the point of measurement (pom) of other authors^55^, corresponds to 1.50 m for the vast majority of trees (93.5% of trees in the last census, 2022). For irregularly shaped trees, due to the presence of bumps, buttresses or aerial roots for instance, the hom is changed depending on the type and level of deformation. For slightly irregular trees, the girth can be measured 20 cm below and up to 50 cm above the deformation (0.7% of trees in the last census), while for extremely irregular trees, the hom was raised at 4.50 m in 2008 (5.5% of trees) to avoid bias in growth estimates due to several hom raising over the long term^55^. For the very large and extremely irregular trees that cannot be measured even at 4.50 m (0.3% of trees), the diameter is measured with the Bitterlich relascope every five years and the value is reported for the subsequent years up to the next measurement. The hom is painted on the trees, in red for the trees belonging to the 36 valuable timber species or species complexes recognized at installation, and in yellow for all other trees (Fig. 2a).

Finally, it has to be noted that the trees with a girth greater than 250 cm between 1982 and 1990 were not measured but attributed to 10-cm wide diameter classes. Since 1991, the girth of these trees has been measured in the field as for the other trees, and converted into diameters in the database.

#### Botanical identification

Three types of botanical determination are available and provided in the data: (1) the vernacular name and the associated scientific name according to the field identification of F. Freytet and E. Dobo in 1991 and of B. Teyssendier de la Serve and J-B. Bélé in 1992; (2) the scientific name according to the identification of D. Beina in 2010; and (3) the scientific name according to the identification of J-F. Gillet in 2011. All names are linked to the African Plant Database maintained by the “Conservatoire et Jardin Botaniques” of Geneve^56^ so that to ensure cross-site comparisons, and address different facets of biodiversity (i.e., functional and phylogenetic).

During the first decade, the identification was limited to 36 valuable timber species or species complexes (Table 2) that have a high or secondary commercial value^1^. The category A was assigned to 15 species of interest for the international market, while the category B was assigned to 21 species or genera of interest for the local market. All other trees and species were considered as category C. The botanical identification of trees belonging to category C was complemented between 1991 and 1995^44,57,58^. A good correspondence was found between the local name in the Issongo language and the scientific name, leading to a total of 240 species or species complexes with herbarium specimens collected for the vast majority of taxa. This reference still used by the field crew, has been conserved up to the last inventory in 2022 and assigned to the trees monitored since 1982 when possible. The botanical identification was subsequently improved in 2010 within the PhD thesis of D. Beina^59^ (n = 321 species, n = 21,854 trees) and during a field mission of J-F. Gillet in 2011 (n = 230 species, n = 3,039 trees), both of them were assisted by the field crew and J-G. Kiki (a temporary field worker involved in the re-census of the plots who has an excellent knowledge of the local tree flora). This information is only available for the trees alive during these two years. Many herbarium specimens were collected by D. Beina and were stored at the “Centre d’Études et de Recherche en Pharmacopée et Médecine Traditionnelle Africaine” in Bangui.

**Table 2:** List of the 36 valuable timber species or species complexes with a high or secondary commercial value recognized at the installation of the M’Baïki PSPs.

Species	Valid name	Commercial name	Code vernacular	Issongo name
**Category A**				
*Entandrophragma cylindricum*		Sapelli	01	Mboyo
*Entandrophragma utile*		Sipo	02	Bokoï
*Entandrophragma candollei*		Kosipo	03	Kanga-Bona
*Entandrophragma angolense*		Tiama	04	Kanga
*Afzelia africana*		Doussié	05	Mokala
*Khaya anthotheca*		Acajou	06	Dèkè
*Lovoa trichilioïdes*		Dibétou	07	Mboyo-Kondi
*Chlorophora excelsa*	*Milicia excelsa*	Iroko	08	Mokoko
*Pterocarpus soyauxii*		Padouk	09	Tola
*Nauclea diderrichii*		Bilinga	10	Kido
*Autranella congolensis*		Mukulungu	11	Bouanga
*Morus mesozygia*		Difou	12	Bonde
*Lophira alata*		Azobé	13	Ngolo
*Erythrophleum guineense*	*Erythrophleum suaveolens*	Tali	14	Kassa
*Guarea cedrata*	*Leplaea cedrata*	Bossé	15	Bombolo-Bombolo
**Category B**				
*Triplochiton scleroxylon*		Ayous	16	Cepa
*Terminalia superba*		Limba	17	Nganga
*Piptadeniastrum africanum*		Dabéma	18	Mokoungou
*Picnanthus angolensis*		Ilomba	19	Kolo
*Ceiba pentandra*		Fromager	20	Bouma
*Ricinodendron heudelotii*		Essessang	21	MBoboko
*Staudtia stipitata*	*Staudtia kamerunensis*	Niové	22	Molanga
*Canarium schweinfurthii*		Aiélé	23	Fatou
*Alstonia congensis*		Emien	24	Mogouga
*Sterculia oblonga*	*Eribroma oblongum*	Eyong	25	GBoyo
*Diospyros crassiflora*		Ebène	26	Bingo
*Oxystigma oxyphyllum*	*Prioria oxyphylla*	Tchitola	27	Ngoulou
*Nesogordonia papaverifera*		Kotibé	28	Molo-Fongoli
*Fagara lemairei*	*Zanthoxyllum lemairei*	Olon	29	Bolongo
*Aningeria sp*.	*Pouteria sp*.	Aniégré	30	MBoulou
*Mammea africana*		Oboto	31	Bolélé
*Ongokea gore*		Angueuk	32	Gbana
*Petersianthus macrocarpus*		Essia	33	Mossoba
*Antiaris africana*		Ako	34	Mongodou
*Funtumia elastica*		Pri	35	Mondembo
*Cola nitida*		Colatier	36	Mako

The category A corresponds to species of interest for the international market, while the category B corresponds to taxa of interest for the local market^1^.

#### Demographic census

During each inventory campaign, missing trees in the 1-ha subplots are systematically checked and the mortality reason is reported (standing death, primary or secondary treefall). Newly recruited trees, with a gbh greater than 30 cm, are spatially located, according to the coordinates of a neighbouring tree and/or the (sub)plot borders. These trees are numbered, measured, and determined botanically as described above. Despite trees being recruited in the field from 30 cm gbh (9.5 cm dbh), the recruitment date in the database corresponds to the date after which dbh is greater than 10 cm.

### Data quality control

#### Data checks and field controls

Since the start of the inventory campaigns in the M’Baïki PSPs, a special effort has been made to control the quality of the data. This control was automated with digital encoding in the field using a HUSKY hand-held computer from 1998 to 2017 (Fig. 2a) and Android-based touch-screen tablets since 2018^60^. The systematic checks made in the field concerned anomalies in girth/diameter values and missing trees.

After the inventory campaign, the same systematic search for anomalies is carried out by comparing the new values with those of the previous inventory campaign. Girth are converted into diameters and the following checks are made: (1) inconsistency for living trees, such as excessive growth (a diameter increment greater than 2 cm.yr^−1^) or decline (diameter increment less than −0.6 cm.yr^−1^), and presence of a re-born tree (previously considered as dead); (2) unauthorised values, such as a diameter inferior to 10 cm for recruited trees, or doubtful values, such as a diameter that increases between the two campaigns even though the hom has been raised; (3) missing data. A selection of anomalies is then sent to the field crew for control and recensus in the field before the final integration into the general database, which was developed using postgreSQL software in 2010 and has been maintained at CIRAD since that.

#### Data processing

Raw data are kept in the database but several correction procedures are applied in the case of hom raising, missing data, or re-born tree.

In order to correct for technical diameter reduction due to hom raising, a simple taper ratio corresponding to the diameter at time *t* measured at raised hom over the diameter at time *t* − 1 measured at original hom. The subsequent diameters from *t* + 1 measured at raised hom are then converted into equivalent diameter at original hom using this ratio. The hom raising can occur several times during the monitoring period, for instance a tree *i* was first measured at 1.50 m then at 3.50 m and finally at 4.50 m since 2008 when all hom raised were standardized to 4.50 m. To take into account successive hom raising, the taper correction is sequentially applied.

Missing data can occur because the tree was recorded in the field but the diameter was not reported or because the information is not available and the tree is fully missing. For the latter, an empty inventory campaign is first created. Then, all missing data are treated similarly with different solutions depending on the sign of the diameter increment between valid measurements (>0 or <0) and whether the hom was raised or not in the field. In the case of a positive diameter increment, missing diameter data are replaced with a linear interpolation. In the case of a negative diameter increment and a hom raising, the procedure with the taper ratio described above is computed between the valid measurements at different hom. Finally, in the case of a negative diameter increment which does not correspond to a hom raising, the last valid diameter value is reported. This type of correction only concern a few trees, that are usually re-born trees. Indeed, some trees can be recorded as dead for few successive campaigns before to be recorded again, and considered as re-born. These peculiar missing data are replaced using the diameter at the re-born date.

In addition, strong inconsistencies in the time-series are checked individually, based on the time-series of tree diameter and tree diameter increment. For the whole 1982–2022 period, an expert-based correction was made a posteriori by S. Gourlet-Fleury, and then the other procedures associated with hom raising or gap filling were run again integrating the expert-based diameter value within the time-series.

## Data Records

### Data set files, format and encoding

The data sets for the control plots are downloadable without restriction as a single archive from CIRAD’s public repository^61^. This archive contains a total of eight csv files for tabular data with comma separating values and two geojson files to store the spatial data with attribute–value pairs and arrays. The character encoding is ASCII text for all files.

The tabular data provided consists of (1) the tree information, including location in the plot, botanical identification and the taxonomic code (tree.csv); (2) the taxonomy used, including the binomial name with authority (taxonomy.csv); (3) the correspondence between vernacular names in Issongo and the species names associated (vernacular.csv); (4) the grouped information on the silvicultural treatment (trees_context.csv); (5) the repeated girth measurements and associated height of measurements (hom) on trees located in the PSPs and the date and cause of mortality for dead trees over the 1982–2022 period (measures.csv); (6) the information on the inventory campaigns (inventories.csv); (7) the description of the observation codes including information on the tree shape and presence of termites/lianas or any damages (observation_codes.csv); and (8) the description of the mortality codes (mortality_codes.csv).

The spatial data consists of (1) the spatial coordinates and description of the 4-ha plots (plots.geojson) and (2) the spatial coordinates and description of the 1-ha subplots (subplots.geojson).

### Variable information

**Fig. 3:**
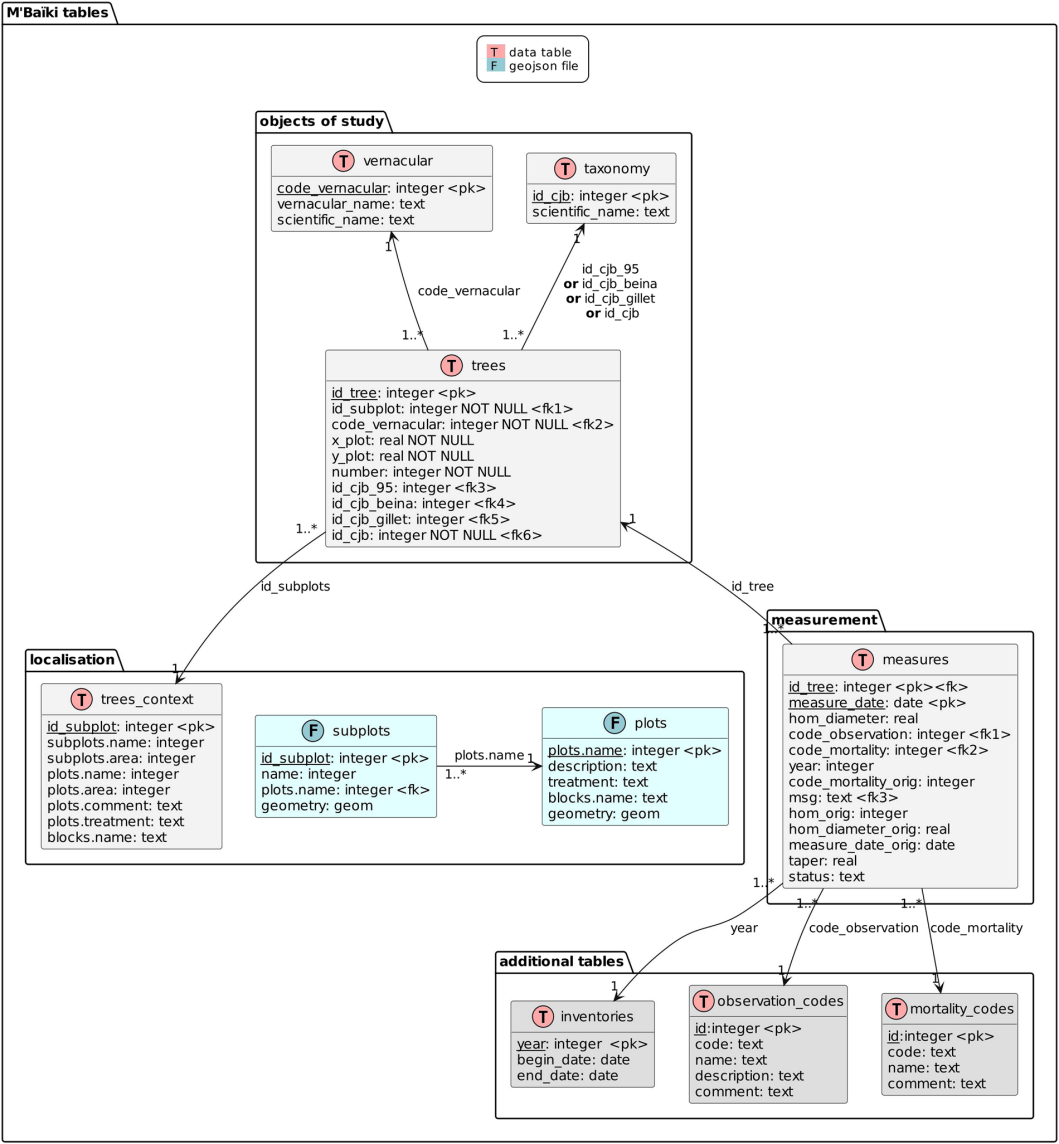
Structure of the general database for the M’Baïki PSPs data, with relationships between the eight data tables (in grey) and the two geojson files (in blue).

The data model relating the eight data tables (in grey) and the two geojson files (in blue) is shown below (Fig. 3). The data model, variables and codes list are described in the data dictionary (cf. data_dictionary.pdf) available for download in the same archive from CIRAD’s public repository^61^.

Data is structured according to the rules of the relational database model (tables and inter-table links). Each table contains a list of variables (corresponding to the columns in the data table), their type (integer, real, text, boolean, date, etc.) and, for some of them, non-nullity integrity constraints. Tables also have a primary key (uniquely identifying each row in the table) and, in some cases, one or more foreign keys (reference to the primary key of another table). The links between tables are labelled with the foreign keys used and the cardinality (“1” row in one table being referenced by 1 to several rows “1..*” in the other table).

## Technical Validation

The data of the M’Baïki PSPs has been earlier used to fit growth and population models^62,63^, to study the dynamics of species composition^40^ and the effect of silvicultural treatments on the diversity of trees^64^ and other life-forms^54^, and to estimate aboveground biomass stocks and sequestration^16,65^. Here, we show the forest structure dynamics and response of tree demography to the silvicultural treatments.

### Forest dynamics

Plot data are classically used to characterize forest structure at the 1-ha subplot scale and various structural attributes can be computed. Here, we used the long term data in the M’Baïki PSPs, with an almost yearly monitoring since 1982, to show the changes in tree density (the number of trees per hectare, N) and the basal area (the sum of the cross-sectional areas at breast height, G, in m^2^ha^−1^) in the control plots and in response to the silvicultural treatments (logging, and logging plus thinning) applied in 1986.

**Fig. 4:**
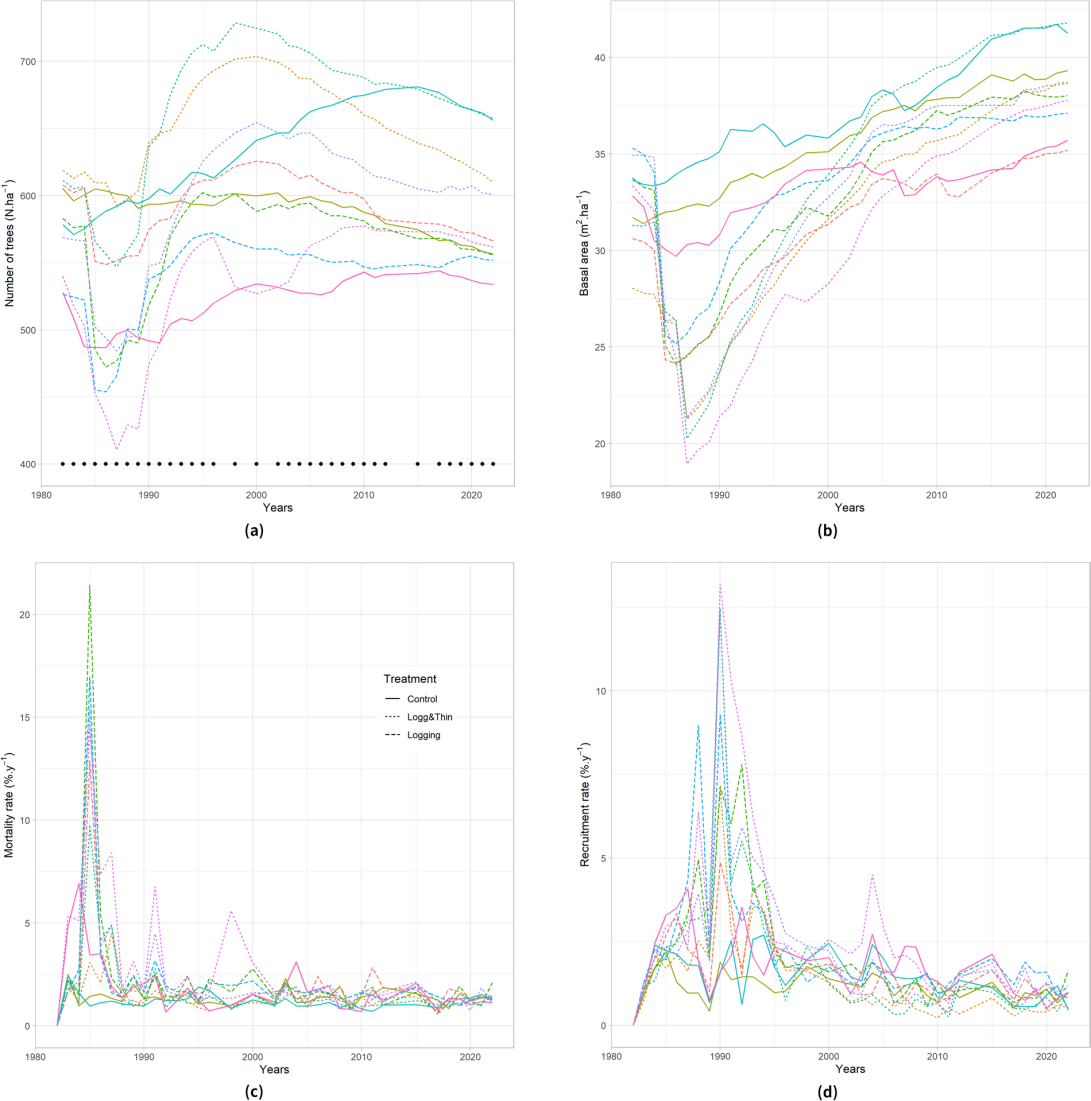
Dynamics of forest structural attributes (stem density (**a**), N in ha^−1^ and basal area (**b**), in m^2^ ha^−1^) and demographic rates (mortality (**c**) and recruitment (**d**), both expressed in number of trees %yr^−1^) and computed at the scale of 4-ha plots with a color code and line type according to the experimental blocks and silviculural treatments, respectively. The 35 inventory campaigns are indicated with dots on (**a**).

In the control plots, the basal area increased over time (Fig. 4a,b) confirming the trends already observed of increasing aboveground biomass and carbon stocks in intact forests across tropical Africa and explained by CO2 fertilization^25^. This increase of forest biomass/carbon stocks is particularly evident in the M’Baïki control plots as already demonstrated for a 24-yr period^64^ and might be also related to forest ageing since the plot installation. The populations from the villages nearby the plots (Fig. 1) have indeed been asked to avoid the plots for their activities (harvest of wood fuel and non-forest timber products).

Both tree density and basal area sharply dropped in the most disturbed plots in the inventory campaigns of 1986 and 1987, and started recovering afterwards, until reaching the initial values in 2000 and 2010, respectively, and even greater values in the last census in 2022 (Fig. 4a,b). Though the noticeable increase of the proportion of pioneer species in the stand, notably due to the recruitment of pioneers after disturbance, the silvicultural treatments had only little impact on the floristic composition of the subplots as quantified 7 to 23 years later^40^ and on the tree diversity as quantified 24 years later^64^.

The data of M’Baïki PSPs are of extreme importance to study the structural complexity of tropical forests and were earlier used to develop a null model for the structure of tropical forests^66^. Previous studies also showed the response of aboveground biomass (in Mg.ha^−1^) to silvicultural treatments^16^ and made it possible to disentangle the contribution of small *versus* large trees to biomass/carbon stocks and sequestration^65^. In contrasts to other sites, yearly measurements are made in M’Baïki PSPs and this can introduce disturbance locally, but the impacts of research activity (trails) on tree growth, species diversity and composition have been shown to be limited^53^.

### Tree demography

PSP data are classically used to describe tree demography. Tree growth is of great interest to evaluate the sustainability of management strategies for productive forests^62^ and also model sustainability under climate changes^67^, for describing ecological strategies^68,69^ and for comparing growth conditions across sites^63^. Recruitment and mortality rates are equally important but are more difficult to estimate due to the relative rarity of these demographic events^70^.

Here, we used the long term data in the M’Baïki PSPs, with an almost yearly monitoring since 1982, to estimate tree recruitment and mortality rates (both in %.yr^−1^). Mortality and recruitment rates in the control plots at M’Baïki fluctuated around 1%/yr (Fig. 4c,d), which is in the range of standard turnover for tropical forests^70^. The net balance between recruitment and mortality in control plots was slightly in favour of a net demographic increase, as reflected by the long-term increase in tree density in the control plots (Fig. 4a).

Disturbance induced by logging, and logging plus thinning (disturbance at the 1-ha subplot was earlier estimated as the % basal area lost^16,40^), strongly affected mortality and recruitment rates. Disturbance immediately resulted in a mortality rate of 15–20% that corresponded to the harvested trees, to the trees impacted by logging damages, and to the poisoned trees (thinning). Delayed mortality due to trees wounded during logging also occurred for 5–10 years^71^. Logging boosted recruitment due to the opening of the canopy in logging gaps. Nonetheless, the peak of recruitment (1992) was delayed with respect to the peak of mortality (1986). It occurred five years after logging and reached 10%. Moreover, the peak of recruitment in disturbed plots was wider than the peak of mortality, *i.e*., the effect of logging on recruitment lasted longer than the effect of logging on mortality. As a consequence, the net balance between recruitment and mortality was strongly negative immediately after logging for a few years, and subsequently positive for the next 10–15 years (results not shown).

Logging is one type of disturbance among others. The transient dynamics observed in the logged plots of M’Baïki are typical of post-disturbance dynamics, while the dynamics of control plots characterise the initial and steady state to which disturbed plots are expected to return on the long term^39^. These control plots are, however, not so stable and show an increase in stand density (in terms of tree density and basal area, Fig. 4a,b, and aboveground biomass^16^).

## Usage Notes

## Data Availability

The R code developed for processing the data (“computed_data.R”) and analysing the forest structure dynamics and the response of tree demography to the silvicultural treatments is available for download in the same archive from CIRAD’s public repository^61^.
